# Disrupted synapses: prefrontal cortex-reward circuit dysfunction in stress-induced depression-like behaviors

**DOI:** 10.3389/fncel.2026.1843871

**Published:** 2026-05-20

**Authors:** Ashraf Mahmud, Mehmet Akif Karaman

**Affiliations:** Liberal Arts Department, American University of the Middle East, Egaila, Kuwait

**Keywords:** chronic stress, depression-like behaviors, major depressive disorder, neuroimmunology, prefrontal cortex, reward circuitry, social defeat

## Abstract

Major depressive disorder (MDD) is a multifactorial, circuit-level disorder often triggered by chronic stress, which fundamentally disrupts the neural networks governing reward processing. Central to this pathology is the prefrontal cortex (PFC), an integration hub exerting top-down executive control over subcortical regions. Here, we synthesize translational and preclinical evidence detailing how chronic stress induces structural, functional, and molecular maladaptations within the PFC and its reward-related downstream projections. By dissecting specific neural pathways—including the PFC’s connections to the nucleus accumbens (NAc), ventral tegmental area (VTA), ventral hippocampus (vHIPP), and lateral habenula (LHb)— we map how projection-specific dysregulation drives distinct depressive phenotypes. Furthermore, we examine the cellular mechanisms underlying these circuit alterations, emphasizing the roles of disrupted neuromodulation (dopamine, glutamate, and serotonin), impaired synaptic plasticity, and robust neuroinflammatory cascades. We highlight notable sex-dependent findings where relevant, illustrating how specific transcriptomic, morphological, and circuit-level responses can diverge between males and females. Finally, we discuss the necessity of moving beyond simplistic behavioral dichotomies and integrating multimodal neurobiological approaches. Ultimately, delineating these precise, circuit-specific vulnerabilities provides a critical framework for developing targeted therapeutics for stress-induced affective disorders.

## Introduction

Major depressive disorder (MDD) is a multifaceted disorder with diverse symptoms showing profound individual variability, often preceded by chronic stress that disrupts neural circuitry (Pizzagalli, 2014; Heshmati and Russo, 2015; Fischer et al., 2016; Belleau et al., 2018). Within this circuitry, the prefrontal cortex (PFC) demonstrates significant evolutionary expansion, particularly in humans and primates, due to the development of the dorsolateral PFC and the frontal pole—structures absent in rodents (Wise, 2008). Primate PFCs are “granular” (featuring a distinct layer IV), whereas rodent PFCs are “agranular” (lacking layer IV); nonetheless, rodent PFCs share “class-common” functions and behaviors with those of primates, such as the temporal organization of goal-directed actions (Carlén, 2017). The rodent medial PFC (mPFC) functions as a complex integration hub composed of diverse projection neurons and interneurons that process long-range signals related to behaviors and cognition while simultaneously interacting within a precise local network of excitatory and inhibitory connections (Anastasiades and Carter, 2021). While the rodent PFC consists of medial, orbitofrontal, and cingulate areas and lacks a direct anatomical homolog of the primate dorsolateral PFC, its prelimbic and infralimbic subregions are considered functionally homologous to the human pregenual and subgenual Anterior Cingulate Cortex (Brodmann Areas 24 and 25), both of which are extensively implicated in MDD (Uylings et al., 2003; Kolb et al., 2012; Kolk and Rakic, 2022; Pizzagalli and Roberts, 2022).

The PFC governs complex cognition and executive functioning, including cognitive control, decision-making, long-term planning, and top-down processing. Furthermore, it is essential for emotional regulation, reward learning, motivated and goal-directed behaviors, and maintaining internal representations of the world (Wise et al., 1996; Miller and Cohen, 2001; Fuster, 2002; Uylings et al., 2003; Dalley et al., 2004; Alexander and Brown, 2011; Euston et al., 2012; Kolb et al., 2012; Starkweather et al., 2018; Wang et al., 2018; Kolk and Rakic, 2022). Because the PFC undergoes a protracted maturation period extending well into young adulthood, this region is highly likely to be susceptible to stress-induced structural and functional alterations (Sowell et al., 1999; Gogtay et al., 2004; Parker et al., 2020; Goodpaster et al., 2026).

Stress is a strong predictor of the onset, maintenance, and severity of MDD. Epidemiological studies indicate that stressful life events precede roughly 80% of depressive episodes and are linked to clinically poor outcomes, including increased symptom burden, more frequent relapse, and a worse overall prognosis (Ormel et al., 2001; Tennant, 2002). Chronic stress fundamentally alters the organization and function of the PFC, inducing potentially reversible changes in its connectivity with other cortical and limbic networks (Liston et al., 2009; Liu et al., 2017; Hare and Duman, 2020). Key structural consequences of this stress include the disorganization of afferent and efferent PFC circuits, reduced myelination, decreased gray-matter volume, contracted dendritic branching, and diminished synaptic density, thus increasing susceptibility to MDD in humans and related behavioral deficits in animal models of depression (Liston et al., 2009; McEwen and Morrison, 2013; Hare and Duman, 2020). These network-level alterations arise from impaired signaling pathways essential for synaptic transmission, plasticity, and the maintenance of neuronal connections, often driven by dysregulated axonal guidance cues (Chaudhury et al., 2015; Yan and Rein, 2022; Mahmud et al., 2023). Consequently, a state of limited synaptic plasticity, termed “synaptic rigidity,” within affect-regulating regions like the PFC and hippocampus has long been postulated as a primary neurobiological driver of mood disorders, likely underlying the distinct cognitive and learning deficits observed in MDD (Manji et al., 2000; Brown et al., 2025).

In this review, we discuss the chronic stress-induced mechanisms that drive PFC circuit reorganization, exacerbating behavioral deficits in rodent models and MDD severity in humans. Specifically, we explore how alterations in various PFC neurotransmitter systems, particularly those governing reward processing, initiate and sustain the maladaptive effects of adult stress exposure. We also incorporate selected sex-dependent observations where applicable to these specific circuit adaptations. Finally, we synthesize emerging translational evidence from rigorous rodent stress paradigms and clinical human studies to elucidate how disrupted PFC synaptic plasticity and connectivity confer stress susceptibility, detailing the precise cellular and network mechanisms responsible for these detrimental phenotypes.

### Prefrontal cortex-reward circuit disruptions in MDD

Dysregulation of PFC-dependent circuits, particularly within the ventromedial (vmPFC), medial (mPFC), and dorsolateral (dlPFC) regions, is a core feature of human depression and depressive-like states in animal models (Greicius et al., 2007; Salvadore et al., 2010; Dzirasa et al., 2013; Kumar et al., 2013; Ayash et al., 2023). In humans, an MDD diagnosis is consistently associated with decreased gray matter volume in the PFC (Banasr et al., 2021). Postmortem analyses of adults with MDD revealed localized reductions in the number, size, and density of glial cells specifically within the PFC (Miguel-Hidalgo et al., 2010). Furthermore, reductions and alterations in PFC oligodendrocytes (Nagy et al., 2020) suggest that regionally specific glial dysfunction may play a critical role in the disease’s pathology. Dysregulation of dorsolateral PFC (dlPFC) activity is often associated with abnormal reward processing (Keedwell et al., 2005; Morgan et al., 2013). Meta-analysis indicates that dysregulated corticostriatal connectivity may broadly underlie these reward-processing deficits in MDD (Ng et al., 2019).

**Figure 1:**
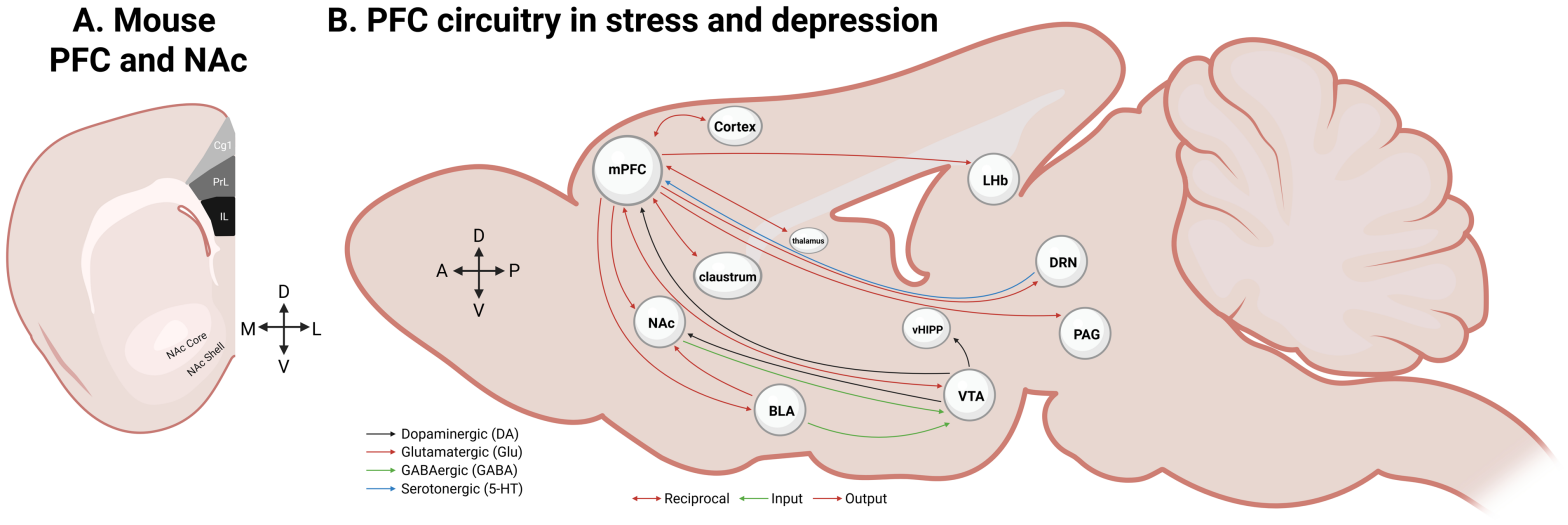
Schematic representation of the prefrontal cortex (PFC) circuitry involved in depressive-like behaviors. **(A)** A depiction of the rodent PFC and the nucleus accumbens (NAc), key brain regions involved in reward processing and depressive-like behaviors, along the dorsoventral (DV) axis. The PFC is further divided into subregions such as cingulate cortex area 1 (Cg1), a specific subdivision of the anterior cingulate cortex (ACC) within the medial PFC, the prelimbic cortex (PL), and the infralimbic cortex (IL). The NAc can be subdivided into the NAc shell and the NAc core. **(B)** Representation of the rodent PFC neurotransmitter systems and reward circuitry in depressive-like behaviors. The PFC is highly connected with other cortical and limbic brain regions by sending projections (→), receiving inputs (←), or making reciprocal (↔) connections. The black lines indicate dopaminergic (DA) projections, the red lines indicate glutamatergic (Glu) projections, the green lines indicate GABAergic (gamma-aminobutyric acid; GABA) projections, and the blue lines indicate serotonergic (5-HT) projections. For example, the PFC sends glutamatergic projections to the NAc, receives dopaminergic inputs from the VTA, and shares reciprocal glutamatergic connections with the VTA. BLA, basolateral amygdala; DRN, dorsal raphe nucleus; IL, infralimbic; LHb, lateral habenula; mPFC, medial prefrontal cortex; NAc, nucleus accumbens; PAG, periaqueductal gray; PFC, prefrontal cortex; PL, prelimbic; vHIPP, ventral hippocampus; VTA, ventral tegmental area. Note: This schematic is simplified for clarity and not to stereotaxic scale.

Stress significantly compromises the structural and functional integrity of the mesocorticolimbic reward circuit, which comprises the PFC, nucleus accumbens (NAc), ventral tegmental area (VTA), amygdala (AMY), hippocampus (HIP), lateral hypothalamus (LH), lateral habenula (LHb), and dorsal striatum (see Figure 1). Within this architecture, the PFC, NAc, and VTA function as central hubs that integrate excitatory and inhibitory inputs to encode reward salience (Smith et al., 2011). Depressed patients show reduced gray matter volume in affective circuits (limbic and striatal components) and in areas constituting the default mode network (DMN), such as the anterior cingulate cortex (ACC) and anterior medial prefrontal regions (Korgaonkar et al., 2014). In healthy humans, generalizable pleasure is encoded by a distributed mesocorticolimbic circuitry involving the ventromedial PFC (Kragel et al., 2023), a feature disrupted in depression. Because appetitive-learning experiences inherently possess a heightened susceptibility to adaptation or extinction (Bouton, 2004), stress-induced disruptions to this circuitry can severely destabilize reward processing, thereby increasing vulnerability to depressive states.

Depression may arise from a breakdown in top-down excitatory control from the mPFC to subcortical reward-related regions, such as the NAc and amygdala, resulting in the maladaptive processing of rewarding and aversive events (Russo and Nestler, 2013). Chronic stress drives this pathology by disrupting dopamine, serotonin, and norepinephrine neuromodulation and synaptic plasticity, resulting in an excitation/inhibition (E/I) imbalance in the mPFC that disconnects it from reward circuitry and entrenches depressive symptoms (LeDuke et al., 2023). For example, chronic stress reduces the E/I balance and synaptic strength of the rostral prelimbic cortex (PrL), whereas the fast-acting antidepressant (2R,6R)-hydroxynorketamine (HNK) reduces chronic stress-induced passive coping behaviors by reinstating this E/I balance and largely reversing alterations in PrL parvalbumin inhibitory neurons (Fong et al., 2026).

A disruption in the integration of PFC networks may underlie dysfunctional appetitive motivation and the dysregulation of emotional valence in depression. For example, inactivating the left, but not the right, dorsolateral PFC (dlPFC) in marmosets diminishes appetitive motivation and heightens threat reactivity, an effect driven by top-down projections to the pregenual cingulate cortex (Area 32) (Wood et al., 2025). Crucially, the administration of ketamine restored this motivational function by engaging mechanisms within downstream subgenual cingulate targets (Area 25) (Wood et al., 2025). Aligning with these preclinical findings, a recent study in humans demonstrated that negative mood states in MDD are characterized by transient increases in delta-band directed connectivity and a distinct functional imbalance between the left and right prefrontal cortices, supporting a model of depression driven by prefrontal disinhibition and hemispheric asymmetry (Myers et al., 2025). Together, these findings provide a robust translational map for PFC-mediated functional asymmetry in emotion regulation. This framework is further reinforced by clinical evidence showing that transcranial direct current stimulation of the dlPFC significantly alleviates anhedonia in patients with depression (Kong et al., 2023). Furthermore, a recent study showed that, compared to healthy controls, aberrant functional hyperconnectivity in the ventromedial PFC (vmPFC) may serve as a key differentiator between anhedonic and non-anhedonic subtypes of depression (Ma et al., 2024). Functional connectivity between the vmPFC and the left middle orbitofrontal cortex correlated negatively with anhedonia (Ma et al., 2024). This suggests that dysregulation within this specific ventral frontal circuit may actively predict the severity of anhedonic symptoms. The susceptibility of these networks to external stimuli is well-documented; for instance, a brain imaging study found that acute psychological stress in healthy subjects resulted in a significant decrease in reward-related responses in the mPFC, notably without affecting ventral striatal responses (Ossewaarde et al., 2011). Conversely, the targeted upregulation of vmPFC activity via neurofeedback was associated with symptom improvement and a secondary increase in the responsiveness of the ventral striatum to positive stimuli, suggesting a functional restoration of the reward-related circuitry (Linden et al., 2012).

Neuroimaging studies investigating MDD and anhedonia reveal that PFC activity varies significantly across distinct phases of reward processing in humans. During reward consumption (‘liking’), hyperactivation is observed in the vmPFC, the mPFC, and the dlPFC, often accompanied by reduced functional connectivity between the posterior vmPFC and frontostriatal regions (Borsini et al., 2020). A similar trend of mPFC and dlPFC hyperactivation is observed during the anticipation phase (‘wanting’). In contrast, deficits in reward learning are associated with vmPFC hypoactivation, manifesting as blunted neural responses or attenuated prediction error signaling (Borsini et al., 2020). Medial prefrontal/midfrontal hyperactivation and corresponding striatal hypoactivation in response to positive stimuli, including social reward (e.g., happy faces), reward outcomes, and reward anticipation are consistently reported in individuals with remitted depression (Hamilton et al., 2012; Zhang et al., 2013; Goldstein-Piekarski and Williams, 2019). However, medial frontal hypoactivation during positive valence processing, particularly in female patients with high levels of anhedonia is also reported (Mitterschiffthaler et al., 2003). Interestingly, improvements in positive affect observed in depressed patients after two months of antidepressant treatment are positively associated with sustained NAc activity and plasticity in the PFC-NAc circuit (Heller et al., 2013). This circuit-behavior correlation was found to be partially reliant on simultaneous decreases in negative affect (Heller et al., 2013). Consistent with these clinical observations, studies in animal models further validate these findings. Two distinct, adaptable groups of mPFC neurons encode social versus nonsocial rewards differently based on an animal’s biological sex and internal state (Isaac et al., 2024). Intact mPFC activity during the reward phase is required for both male and female mice for appropriate reward-seeking behavior (Isaac et al., 2024). Conversely, persistent hyperactivation of the mPFC suppresses the drive for natural rewards by inducing new brain-wide functional connections that directly predict the severity of an individual’s anhedonia (Ferenczi et al., 2016). Taken together, these findings suggest that dysfunctions in PFC circuitry underlying reward processing fundamentally contribute to the emotional deficits and anhedonia observed in depression.

### Animal models of stress-induced depression-like behaviors

**Figure 2:**
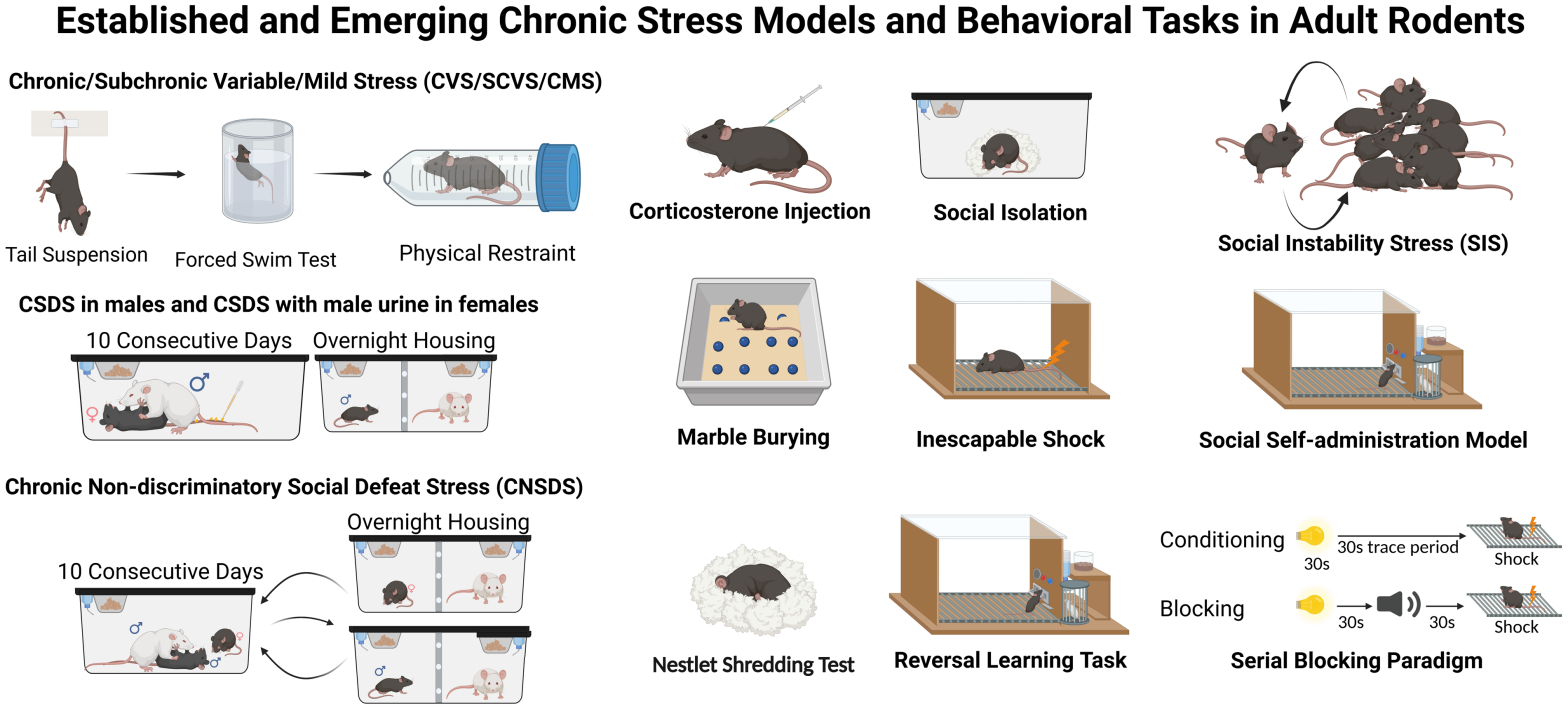
Established and emerging rodent paradigms and behavioral assessments for chronic stress-induced depressive-like behaviors. This figure outlines widely used and emerging paradigms of chronic stress used to investigate stress susceptibility in both male and female rodents and to identify the divergent effects of chronic stress at the behavioral, cellular, and molecular levels. Different variants of chronic unpredictable mild stress (CUMS/CMS), chronic variable stress (CVS) paradigms exist, in which experimental mice are chronically exposed to various stressors such as limited bedding, tail suspension, forced swimming, inescapable shock, and physical restraint in a random order, compared to stress-naïve controls. Alternatively, social isolation and exogenous corticosterone administration reliably increase stress hormones and can be used in both males and females to investigate the endocrinological outcomes of chronic stress. Social instability stress model involves disturbing an animal’s social hierarchy; while often used in males, this paradigm can be highly labor-intensive. A commonly used and validated model of chronic stress is chronic social defeat stress (CSDS), in which experimental mice are exposed to aggressive CD-1 mice during daily social defeat sessions (5–10 min daily for 10–21 consecutive days). In contrast, control animals are housed with a conspecific, separated by a perforated divider that allows for sensory stimuli without physical contact. Following CSDS, the sociability of all mice is assessed in a social interaction test (SIT) using a novel social target. A major strength of this model is that each experimental mouse can be classified into a *susceptible* or *resilient* group based on their SIT sociability score. Research shows that significant behavioral, molecular and transcriptional differences emerge between resilient and susceptible mice, indicating that stress resilience is an active, dynamic neurophysiological adaptation. Because female mice are not readily attacked by male CD-1 aggressors in standard CSDS, variations of the CSDS model such as CSDS with male urine or chronic non-discriminatory social defeat stress (CNSDS) have been developed and are gaining significant traction. To investigate sex differences in stress susceptibility, standard CSDS in males can be implemented in parallel with a modified CSDS protocol with male urine for females. Behavioral tasks such as the marble burying test (evaluating anxiety-like states and repetitive behaviors) and the nestlet shredding test (evaluating self-care-like behaviors) can be used to complement pre- and post-stress behavioral profiling. Finally, novel paradigms, including the social self-administration model, the reversal learning task, and the serial blocking paradigm, can also be utilized to assess complex cognitive, reward processing, and learning deficits induced by chronic stress.

Animal models, particularly rodent models, are foundational to our understanding of the cellular and molecular mechanisms driving depression-like behaviors. These models typically employ prolonged and/or unpredictable stressors such as social stress, physical restraint, inescapable foot shock, pharmacological activation of the stress system, and other environmental stressors (e.g., wet bedding, loud noise) to induce measurable behavioral deficits including social avoidance, learning and cognitive impairments, learned helplessness, changes in appetite and body weight, disruptions in sleep and circadian rhythms, and altered self-care or grooming (Nestler and Hyman, 2010; Czéh et al., 2016; Wang et al., 2017; Mahmud et al., 2023). For example, Chronic Unpredictable Mild Stress (CUMS/CMS), Chronic Variable Stress (CVS), and their variant paradigms expose animals to multiple unpredictable stressors (e.g., damp bedding, altered light cycles), often daily, to induce anhedonia and metabolic dysregulation, capturing the unpredictable, daily hassles experienced by humans. However, these paradigms have high inter-laboratory variability and intense labor requirements. Conversely, Chronic Restraint Stress (CRS) involves repeated physical immobilization and offers high reproducibility and strong construct validity for studying hypothalamic–pituitary–adrenal (HPA) axis dysregulation and structural remodeling, such as the dendritic atrophy observed in the PFC (Nestler and Hyman, 2010; Mahmud et al., 2023). However, animals can habituate to CRS over time. Another prominent paradigm is the chronic social defeat stress (CSDS) paradigm, which allows researchers to segregate animals into “susceptible” or “resilient” groups based on their sociability following repeated social stress exposure (Golden et al., 2011). Because aggressive male conspecifics do not naturally attack females, the standard CSDS model is difficult to implement in females, hindering the study of potential sex differences and underlying mechanisms under identical stress conditions. To overcome this, researchers have adapted different paradigms for females using techniques such as chemogenetic manipulation to force male aggression (Takahashi et al., 2017), swabbing experimental females with male urine to provoke attacks from CD-1 aggressors (Harris et al., 2018), vicarious social defeat stress model (Iñiguez et al., 2018), the chronic non-discriminatory social defeat stress model (Yohn et al., 2019), and *fem*CSDS model (Patel et al., 2026). Figure 2 depicts some commonly used and promising models and behavioral tasks for chronic stress used in adult rodents.

The selection of specific stress models for males and females has significant implications for experimental outcomes. Transcriptomic analyses of PFC and NAc tissues across three distinct adult stress models (CSDS, social isolation, and CVS) reveal substantial divergence in the overall gene expression response. While a small fraction (approximately 25%) of genes is similarly affected across all models, the vast majority of transcriptomic alterations are model-specific, underscoring the fact that different stressors induce unique molecular signatures within key affective circuits (Scarpa et al., 2020). A recent study integrating operant social reward seeking procedure (Venniro and Shaham, 2020) in male and female mice after social stress (social defeat or witness defeat) also found clear task- and -sex-specific behavioral adaptations: while social defeat attenuated social reward seeking specifically in males, witness defeat potentiated seeking behavior selectively in females (Navarrete et al., 2024).

To capture the full complexity of these individual differences and sex-specific alterations, there is a growing need to expand beyond conventional behavioral paradigms. For example, a “cumulative social index score,” derived from aggregated operant metrics, provides a highly robust scoring method offers a more naturalistic understanding of resilience mechanisms. Similarly, composite metrics like the social engagement and the emotionality indices, which rely on machine learning-based tools and comprehensive assessments of multiple behavioral modalities, appear to be effective in capturing individual differences in stress resilience (Yohn et al., 2019; Restrepo-Lozano et al., 2025). Finally, novel and complex *complementary* tasks, such as restaurant row task (Cuttoli et al., 2024), operant reversal learning task (RLT) (Torres-Berrío et al., 2024), and serial blocking effect (Mahmud et al., 2019) can be used to evaluate post-stress outcomes across multiple cognitive domains, including decision-making, motivation, cognitive flexibility, learning, memory, and attentional processes that are commonly altered in depression.

### Chronic stress impairs synaptic plasticity and connectivity within prefrontal reward circuitry

The PFC contains anatomically distinct neuronal ensembles that are selectively recruited based on the emotional valence of an experience, thereby driving different behavioral outcomes. Specifically, positive-valence experiences (like cocaine exposure) preferentially activate an NPAS4 + population in the mPFC, projecting strongly to the NAc, while negative-valence experiences (like shock) activate a separate mPFC population that projects predominantly to the LHb, a key center for aversion (Ye et al., 2016). Importantly, NPAS4 is linked to cellular and microcircuit processes that regulate the balance of E/I ratio, mediating an adaptive plasticity mechanism in which high levels of neuronal excitation trigger the formation of new inhibitory synapses on the excited cells (Spiegel et al., 2014; Ye et al., 2016). A recent study found that mPFC NPAS4 acts as a critical molecular switch that governs reward processing deficits following chronic stress. While NPAS4 is strictly required for the onset of chronic social defeat stress (CSDS)-induced anhedonia, it plays no causal role in the expression of social avoidance or anxiety (Hughes et al., 2023). This behavioral dissociation is driven by NPAS4-mediated structural and functional alterations: it reduces dendritic spine density and blunts excitatory synaptic transmission. The genes upregulated by NPAS4 in CSDS-susceptible animals significantly overlap with the dysregulated transcriptomes identified in the postmortem brains of neuropsychiatric patients including depression (Hughes et al., 2023).

Alterations in PFC reward circuits may also explain the heightened vulnerability to depression observed in females, with gonadal hormones likely playing a critical role. Altered reward processing and learning, particularly at the onset of symptoms, may disproportionately affect women (Whitton et al., 2015; Rupprechter et al., 2018; Halahakoon et al., 2020), and may be impacted by the estrous cycle. Circulating estrogen sensitizes the stress system by enhancing noradrenergic and dopaminergic activity in female rodents (Bangasser and Valentino, 2012), paralleling the condition in humans, where women show reduced expression of COMT, leading to decreased catecholamine catabolism (Tunbridge, 2010; Datta and Arnsten, 2019). While chronic stress is known to influence Pavlovian and instrumental reward learning in males (Xu et al., 2017), a more complex, state-dependent dynamic in reward learning arises in females driven by the estrous cycle. Acute stress facilitates reward learning in males, but in females the effect is bidirectional depending on the estrous cycle: stress enhances conditioned responding in non-estrus females but suppresses it in estrus females (Johnston et al., 2026). However, this cycle-dependent divergence disappears with stress history. By exposing female rats to restraint stress prior to Pavlovian conditioning, the authors demonstrated that females exposed to repeated prior stress exhibited uniformly elevated conditioned responding, regardless of their cycle stage (Johnston et al., 2026). At the cellular level, basolateral amygdala (BLA)-projecting mPFC pyramidal neurons are uniquely sensitive to both the independent and stress-interactive effects of estrogen. Beyond actively modulating the structural stress response, estrogen independently promotes neural plasticity in this circuit by increasing dendritic spine density in unstressed animals (Shansky et al., 2010), highlighting its fundamental role in shaping PFC dendritic architecture. Collectively, these data suggest that stress-induced disruptions in reward learning may be integral to the etiology of depressional states in females, rather than contributing to their long-term maintenance.

By longitudinally tracking CSDS-induced changes in Cg1/M2 cortex pyramidal neurons using two-photon imaging during reward-directed actions, researchers found that stress-induced deficits are not unitary events, but a cumulative continuum wherein repeated exposure progressively impairs both reward processing and individual neuronal function (Barthas et al., 2020). The zinc finger protein, *Zfp189* in prefrontal cortical neurons may orchestrate a transcriptional network for behavioral resilience. Using CRISPR-mediated epigenetic editing, the researchers demonstrated that by recruiting transcription factor CREB to the *Zfp189* promoter boosts resilience, while suppressing the gene with the methyltransferase G9a induces susceptibility (Lorsch et al., 2019). Targeting epigenetic remodeling via the intra-mPFC infusion of MS-275, a selective inhibitor of histone deacetylases (HDACs), was sufficient to induce robust antidepressant-like behavioral responses following CSDS (Covington et al., 2015). Crucially, these data suggest that resilience is not merely the absence of pathology (Han and Nestler, 2017), but a unique, active reorganization of PFC activity distinct from that observed in both susceptible and control mice.

Exposure to uncontrollable stress rapidly takes the PFC ‘offline’ via a potent intracellular signaling cascade (Datta and Arnsten, 2019). During stress, increased release of catecholamines, specifically norepinephrine and dopamine, hyperactivates α1-adrenergic receptors and D1 dopamine receptors (Gamo et al., 2015). This receptor engagement triggers a feedforward calcium-cAMP loop that opens proximal potassium channels, actively suppressing cellular firing and reducing network connectivity (Datta and Arnsten, 2019). When this stress becomes chronic, the sustained physiological silencing physically degrades the network, leading to the atrophy of dendrites and dendritic spines. Indeed, animal models demonstrate that the stress-induced loss of dendritic spines in PFC neurons is directly linked to impaired PFC function and deficits in associated behaviors (Liston et al., 2006; Radley et al., 2006; Dias-Ferreira et al., 2009; Hains et al., 2009; Shansky et al., 2009). We recently showed that following CSDS, PFC pyramidal neurons exhibit a targeted loss of dendritic spines exclusively on their apical, rather than basal, dendrites (Mahmud et al., 2025). We identified the Netrin-1 guidance cue receptor gene *Dcc* (deleted in colorectal cancer) as the critical molecular driver of this pathology. Notably, DCC receptors influence stress susceptibility or resilience by altering the apical dendritic architecture of PFC pyramidal neurons, particularly those projecting to the nucleus accumbens shell (Mahmud et al., 2025). The role of PFC DCC receptors following chronic stress in females requires further investigation and may provide crucial mechanistic insights into sex-dependent stress susceptibility, given that DCC signaling mediates sex differences in other psychiatric conditions (Hernandez et al., 2025).

These effects may stem from mechanisms related to synaptic maintenance and integrity. Downregulation of the long non-coding RNA LINC00473 in the mPFC promotes stress resilience in female, but not male, mice (Issler et al., 2020). LINC00473 and DCC receptors may interact to influence this stress susceptibility (Li et al., 2019; Soutschek and Schratt, 2023). Another potential mechanism involves the modulation of the mammalian target of rapamycin complex-1 (mTORC1) signaling cascade (Agrawal and Welshhans, 2021; Tcherkezian et al., 2021). Postmortem analyses reveal that, compared to healthy controls, levels of the mTORC1 inhibitor REDD1 (regulated in development and DNA damage responses-1) are significantly elevated in the PFC of individuals with MDD (Ota et al., 2014). REDD1 in the PFC is both necessary and sufficient to drive the deficits in synaptic integrity and mTORC1 signaling caused by chronic unpredictable stress. Conversely, the ketamine-mediated reversal of stress-induced synaptic spine loss requires mTORC1 activation in the mPFC (Li et al., 2010). Viral-mediated increases in synapse numbers in the PFC, thus enhancing ketamine’s intracellular signals prevented the behavioral effects of chronic stress (Ota et al., 2014), suggesting that restoring synaptic integrity within the PFC reward circuitry is sufficient to produce an antidepressant effect.

### PFC neurotransmitter systems in chronic stress

Dysregulations such as variations in levels, altered receptor activity, and impaired signaling pathways of neurotransmitter systems such as dopamine (DA), glutamate (Glu), serotonin [5-hydroxytryptamine (5-HT)], and gamma-aminobutyric acid (GABA) within the PFC drive the development of depressive-like states through the alteration of reward circuitry. The mPFC critically mediates the effects of fast-acting antidepressants (e.g., ketamine) and psychedelics by stabilizing these reward circuits. Research shows that while silencing mPFC neurons blocks ketamine’s effects, microinfusion of ketamine into IL-PFC, or optogenetic activation of CaMKII2α-expressing pyramidal neurons in the IL-PFC reproduces them, an effect robustly associated with increased synaptic plasticity (Fuchikami et al., 2015). Psychedelics like psilocybin rewire large-scale cortical networks, notably the mPFC, inducing the activity-dependent neuroplasticity required for lasting psychological and behavioral change (Jiang et al., 2026; Siegel et al., 2026). Studies in both humans and animal models of depression show that the degree of antidepressant efficacy may depend heavily on a drug’s ability to alter PFC DA signaling. For example, chronic antidepressant treatment including fluoxetine stimulates DA release in the PFC/NAc while inhibiting dopaminergic function in the VTA by enhancing the synaptic levels of 5-HT, thus alleviating states related to DA dysfunction (Prisco and Esposito, 1995; Ichikawa and Meltzer, 1999; Dunlop and Nemeroff, 2007). Recent studies also implicate PFC D2R + neurons in modulating depressive-like behaviors and mediating the effects of mechanistically distinct antidepressants, such as ketamine and fluoxetine, often in a precise, pathway-specific manner (e.g., mPFC-VTA and mPFC-NAc projections) (Wilke et al., 2022; Chen et al., 2024).

Dopaminergic signaling in the mPFC appears particularly critical for initiating and maintaining the behavioral effects of rapid-acting antidepressants. PFC DA actions are highly cell-type-, region-, and layer-specific, dictated by precise network wiring. Using double *in situ* hybridization, researchers assessed the expression of D1 and D2 receptor messenger RNAs in pyramidal neurons and GABAergic interneurons in rat PFC and found that although a larger proportion of GABAergic neurons express D1 receptors, the overall number of D1 positive cells is higher in the pyramidal population due to their greater abundance (Santana et al., 2009). Cells expressing the same dopamine receptor transcript are rarely positioned near each other, yet D2-positive pyramidal neurons are frequently found adjacent to GABAergic cells that lack the D2 receptor (Santana et al., 2009). Following chronic unpredictable stress (CUS), mPFC levels of β2 adrenoceptors and D1 receptors were reduced in male rats, but not in females (Jankovic et al., 2022). Optogenetic stimulation of mPFC neurons expressing D1 receptors (but not D2 receptors) induced enduring antidepressant-like effects, whereas the pharmacological blockade of D1 receptors prevented ketamine’s rapid antidepressant action in rats. These effects are dependent on the recruitment of the mPFC-BLA circuit (Hare et al., 2019). Ketamine-induced mPFC spinogenesis is also driven by D1 receptor activation and requires postsynaptic protein kinase A (PKA) activity (Wu et al., 2021a). Notably, chemogenetic blocking of local PFC dopamine release or VTA dopaminergic transmission is sufficient to prevent ketamine’s action in the mPFC (Wu et al., 2021a, 2021b).

Glutamatergic signals in the PFC regulate depression-like behaviors by stabilizing neural circuits, synaptic plasticity, and neuronal excitability. Particularly, GluN2D subunit-containing N-methyl-D-aspartate receptors (NMDARs) located on GABAergic interneurons in the PFC appear as a promising new therapeutic target. Selectively inhibiting these GluN2D-containing NMDARs suppresses GABAergic output, leading to an enhancement of excitatory neurotransmission (i.e., pyramidal cell disinhibition) and rapid antidepressant action in both stress-naïve and CSDS-exposed mice (Zhang et al., 2025). These effects were abolished when the *Grin2d* gene was globally knocked out or selectively deleted in parvalbumin-positive interneurons in the PFC, a process that involved the mTOR signaling pathway (Zhang et al., 2025). Preclinical and clinical studies reinforce the idea that glutamatergic dysfunction, particularly, in PFC astrocytes contribute to susceptibility to depressional states (Fan et al., 2023a; Wada et al., 2024). Chronic stress induces sex-specific astrocytic remodeling, specifically, atrophy in males and hypertrophy in females, which is regulated in part by gonadal hormones. In females, these stress-induced effects on microglia are distinctly estradiol-dependent (Bollinger et al., 2019). Chen et al. (2025) identified that the downregulation of the endoplasmic reticulum stress sensor PERK in PFC astrocytes contribute to depression-related phenotypes by inducing dendritic spine loss, pyramidal neuron hypoactivity, and weakened PFC functional connectivity (Chen et al., 2025). Overall, chronic stress decreases glutamate neurotransmission in the PFC (Duman et al., 2019).

Considerable alterations occur across PFC neurotransmitter systems during chronic stress. These changes lead the PFC to undergo distinct pathway-specific and sex-specific functional, morphological, and transcriptional remodeling. In the following section, we summarize recent findings implicating PFC connections with other brain regions, particularly those involved in reward processing, and detail how chronic stress affect these pathways to initiate and maintain depression-like behaviors.

### The PFC-NAc circuit

Chronic stress disrupts the NAc, a key limbic region involved in reward learning and processing, motivation, and hedonic tone, often by attenuating dopaminergic activity and impairing synaptic strength and plasticity (Lemos et al., 2012; Spring et al., 2021; Zhang et al., 2024; Lucantonio et al., 2025). The mPFC-NAc pathway plays a critical, synergistic role in reversing the behavioral abnormalities induced by chronic stress. For instance, the combined application of chronic venlafaxine administration and repeated PrL-NAc optogenetic stimulation is required to fully reverse chronic mild stress (CMS)-induced behavioral and cognitive deficits, as neither treatment alone is effective (Papp et al., 2023), suggesting that excitatory input from the PFC is critical for ameliorating the effects of chronic stress within the NAc. Bittar et al. (2021) showed that the transcriptional response to chronic variable stress is highly distinct between sexes within these specific projections. In NAc-projecting neurons, females show increased expression of plasticity-related genes like *Grin1*, *Grin2a*, and *Shank1*, whereas males do not exhibit these specific increases (Bittar et al., 2021). Conversely, in VTA-projecting neurons, females show broad increases in GABA and glutamate receptor genes (*Gabra1*, *Grin2a*), whereas males show a mixed profile with several genes downregulated. Morphologically, stress induces a severe reduction in dendritic complexity specifically in VTA-projecting mPFC neurons in males, compared to females. Functionally, females exhibit an increased frequency and amplitude of excitatory postsynaptic currents in NAc-projecting neurons, a change absent in males. Crucially, this functional hyperactivity in the corticoaccumbal pathway appears to drive depression-like behaviors in females, as chemogenetic inhibition of these neurons successfully rescues the phenotype in females but not in males (Bittar et al., 2021). Together, these findings suggest that while behavioral outcomes may appear similar, the underlying transcriptional responses and neural circuit alterations in response to chronic stress diverge significantly between sexes, underscoring the critical need for sex-specific treatment strategies.

### The PFC-VTA circuit

Chronic stress significantly alters VTA GABA, glutamate, and dopamine neurons, which in turn regulate behaviors related to anhedonia (Holly and Miczek, 2016; Lowes et al., 2021; McGovern et al., 2024). Compared to age, sex, and handedness matched healthy controls, the key symptoms of anhedonia in individuals with depression was associated both with the structural deficit (reduced superolateral medial forebrain bundle volume) and the functional change (increased VTA–PFC hyperconnectivity) (Bracht et al., 2022). This suggests that the observed VTA–PFC hyperconnectivity may reflect a maladaptive or compensatory response related to anhedonia.

The mPFC appears to exert top-down regulation over VTA DA neurons and stress disrupt this regulation. For instance, increased dopamine D2 receptor dimerization is observed in the PFC of adult susceptible mice compared to controls (Bagalkot et al., 2015). Using single-unit extracellular recordings, researchers evaluated the impact of IL PFC versus LHb modulation on VTA dopamine neurons following 5–7 weeks of CMS. Stimulating the IL PFC specifically targeted the medial VTA neurons that are vulnerable to CMS, whereas LHb stimulation targeted the stress-resilient lateral VTA. Furthermore, only selective inactivation of the IL PFC, but not the LHb was sufficient to rescue dopamine population activity in stressed animals (Moreines et al., 2017). These findings suggest that mPFC acts as the primary upstream driver of the diminished dopamine response observed in models of depression-like behaviors.

The VTA dopamine projections exert opposing controls over stress susceptibility depending on their target region. Specifically, phasic optical activation of the DA VTA-NAc pathway induces susceptibility to social defeat stress, whereas its inhibition confers resilience (Chaudhury et al., 2013). In contrast, the VTA-mPFC projection appears essential for maintaining resilience: its activation does not induce susceptibility, yet its inhibition promotes a susceptible phenotype (Chaudhury et al., 2013), suggesting a cell- and circuit-specificity in susceptibility to social stress. Using unpredictable CMS, Liu et al. (2018) investigated the output-specific regulatory roles of VTA dopamine neurons in nociception and depression-like behaviors, and found that CMS selectively decreased the firing activity of VTA-mPFC neurons, but not VTA-NAc neurons. Crucially, this study revealed a molecular and circuit segregation: the relief of depressive-like behaviors was dependent on blocking brain-derived neurotrophic factor (BDNF) signaling in the mPFC, whereas the nociceptive response was regulated by BDNF signaling in the NAc shell (Liu et al., 2018). The PFC also serves as a critical site for sex- and region-specific epigenetic modifications, such as DNA methylation of the *Bdnf* promoter and the astrocyte-specific *TrkB. T1* promoter. These differential changes significantly impact glutamatergic and GABAergic neurotransmission, brain development, and stress reactivity (Hodes et al., 2017). Chronic stress reduces BDNF expression in the PFC and disrupts a critical activity-dependent pathway where BDNF normally binds to TRKB receptors to activate AKT and ERK kinases, which are essential for stimulating the mTORC1 signaling pathway (Sanacora et al., 2022). Consequently, the deactivation of this BDNF-mTORC1 axis leads to diminished glutamate release and impaired synaptic transmission within the PFC, a process further evidenced by the distinct epigenetic and transcriptional regulation of BDNF observed in CSDS-resilient versus susceptible mice (Mallei et al., 2019). Together, these findings illuminate projection-specific mechanisms within the mesolimbic reward circuitry, indicating that the VTA-PFC pathway actively controls depressive-like phenotypes.

### The PFC-BLA circuit

Historically linked primarily to anxiety, the BLA, a key brain region involved in emotional processing receives direct inputs from the PFC. Recent findings suggest that the mPFC–BLA circuit regulates sex and age-specific aspects of chronic stress-induced depression-like behaviors. Chemogenetic stimulation of PFC-amygdala circuitry ameliorated CSDS-induced social interaction deficits and restored PFC-dependent limbic synchrony in stress-susceptible animals (Hultman et al., 2016). These effects likely result from attenuated BLA theta oscillations and increased exploratory behavior (Guo et al., 2025). Interestingly, inactivation of BLA prior to chronic stress prevents learning impairment and astroglial loss in the PFC (Tripathi et al., 2019), highlighting a viable clinical strategy (e.g., inhibitory transcranial magnetic stimulation, targeted neuromodulation) for ameliorating stress-induced PFC dysfunctions.

Signaling within the mPFC-BLA pathway may promote stress resilience through the generalization of reward. Activation of the IL projection to calbindin-1 + neurons in the posterior BLA allows mice to generalize learned rewards to ambiguous cues following reward conditioning, and reverses both anxiety- and depression-like behaviors caused by chronic stress (Yu et al., 2022). The PFC glutamatergic neurons maintain emotional homeostasis by exerting persistent inhibition over BLA inhibitory interneurons. Under stressful conditions, BLA dopamine levels elevate and the PFC’s regulatory control is dampened (Rosenkranz and Grace, 2001; Lamanna et al., 2022), resulting in the disinhibition of sensory-driven affective responses. Together, these studies highlight that stress-induced circuit dysfunctions often result from the synergistic dysregulation of multiple interacting neurotransmitter systems. However, the exact etiological and temporal causality of these alterations remains unclear. Whether these network changes represent preexisting biological vulnerabilities that increase stress susceptibility, or are merely the consequence of active neuroadaptational processes, requires thorough investigation utilizing multimodal, multi-method techniques such as intersectional genetics, multiphoton *in vivo* neuroimaging, and multiplex optogenetics.

### The PFC-PVT circuit

Paraventricular thalamus (PVT) is a critical brain area in mediating depression-related behaviors such as reward, sleep, motivation, and stress response (Hsu et al., 2014; Kato et al., 2019; Otis et al., 2019; Li et al., 2024). Bifurcating axons of PVT projection neurons also innervate several other stress-mediating brain areas including the PFC, NAc and amygdala (Krishnan et al., 2007; Cheng et al., 2019; Otis et al., 2019; Yamamuro et al., 2020). Importantly, the mPFC-PVT circuit particularly encodes sociability but not other natural reward (e.g., food consumption) or anxiety in adulthood as chemogenetic or optogenetic suppression of this circuit induced sociability deficits without affecting anxiety or feeding behaviors (Yamamuro et al., 2020). A recent study employed a behavioral subtype-based approach, utilizing the social interaction test (SIT) and sucrose preference test (SPT), within a subchronic and mild CSDS model to identify novel circuit-level molecular mechanisms underlying strain and individual differences in stress susceptibility (Li et al., 2024). Researchers identified four distinct animal subtypes: a resilient subtype (RES; resilient in both the SIT and SPT), a social avoidance subtype (SA; susceptible in the SIT but resilient in the SPT), an anhedonia subtype (ANH; susceptible in the SPT but resilient in the SIT), and an SA: ANH subtype (susceptible in both tests). The mPFC-BLA pathway mediates social deficits, the mPFC-NAc pathway mediates anhedonia, and the mPFC-anterior PVT pathway specifically determines the severe SA: ANH subtype (Li et al., 2024). The precise molecular mechanism driving this SA: ANH phenotype involves the KDM5C-mediated epigenetic repression of the *Shisa2* gene within the specific mPFC neurons that project to the anterior PVT (Li et al., 2024).

Using chemogenetic and electrophysiological approaches, a recent study implicated neuronal nitric oxide synthase (nNOS)-expressing neurons in the mPFC, particularly those projecting to the posterior PVT, as essential regulators of depression-like behaviors (Liang et al., 2025). The activation of these mPFC nNOS-expressing neurons induces the release of nitric oxide and enhances the nitrosylation of cyclindependent kinase 5 which are involved in cognition and synaptic plasticity (Liang et al., 2025). Another recent study found that females display heightened excitability of dorsomedial PFC parvalbumin interneurons (PV-INs), which leads to reduced long-term potentiation at inputs from the mediodorsal thalamus (Zheng et al., 2025). Optogenetic modulation of these PV-INs alters the winner effect, regulating sex-specific competitive behaviors (Zheng et al., 2025). Together, these findings suggest that social defeat and status loss induce depressive-like behaviors by fundamentally altering mPFC-thalamic circuitry. Importantly, these mechanisms may not only underlie sex differences in social competitiveness but could also be leveraged to bolster resilience against the maladaptive sequelae of social stress.

### The PFC-DRN circuit

Chronic stress induces significant structural and functional alterations in the Dorsal Raphe Nucleus (DRN), notably disrupting the synaptic plasticity and activity of serotonergic (5-HT) neurons, altering serotonin release in terminal regions including the PFC, and desensitizing 5-HT autoreceptors (Amat et al., 2005; Rozeske et al., 2011; Natarajan et al., 2017; Al-Kachak et al., 2024; Camargo et al., 2025). Chronic restraint stress triggers a selective loss of p11 (annexin II light chain, S100A10), a multifunctional protein that binds to 5-HT receptors and is highly enriched in D2 + glutamatergic neurons specifically within layer II/III of PrL (Seo et al., 2017). The viral overexpression of p11 specifically in these D2 + PrL neurons is sufficient to restore glutamatergic transmission and rescue depression-like behaviors in both stressed animals and those with genetic p11 deletions (Seo et al., 2017). While this study identifies unique cellular and molecular signatures of chronic stress, the generalizability of these findings across diverse age groups and sexes requires further investigation. Chronic deep brain stimulation of the vmPFC mitigates stress-induced social avoidance by retreating 5-HT hypoexcitability, restoring E/I balance, and reversing stress-induced dendritic changes in the DRN (Veerakumar et al., 2014). These restorative effects appear to occur independently of the mesolimbic dopaminergic reward system (Rea et al., 2013). Notably, the optogenetic silencing of the vmPFC-to-DRN projection specifically during a social defeat episode was sufficient to prevent subsequent social avoidance (Challis et al., 2014). These findings implicate the mPFC–DRN projection as a critical neural substrate for altered motivation under chronic stress.

### The PFC-vHIPP circuit

Dysfunction within hippocampal neuronal circuits can readily induce depression-like phenotypes. Specifically, decades of research confirm several key clinical observations: individuals with MDD exhibit significantly reduced hippocampal volumes compared to healthy controls (Sheline et al., 1996); the degree of this volumetric decline strongly correlates with cumulative disease duration, the number of recurrent episodes, and an earlier age of onset (MacQueen and Frodl, 2011); and crucially, a smaller hippocampus is predictive of poorer clinical outcomes (Sheline, 2011)—an effect that antidepressant medications may partially mitigate (Sapolsky, 2001; Campbell and Macqueen, 2004). The hippocampus shares major reciprocal connections with the PFC. The IL receives direct projections from the ventral hippocampus (vHIPP) in layers II/III and V (Goldman-Rakic et al., 1984; Liu and Carter, 2018). The PFC-hippocampus neural circuit is crucial for emotion regulation and cognition, and shows marked structural and functional deficits in first-episode, medication-naïve adolescents with MDD compared to healthy controls (Geng et al., 2016). These anatomical changes are associated with cognitive deficits, suggesting that aberrant PFC-hippocampal organization is a neuropathophysiological feature present from the earliest stages of MDD.

High density of glucocorticoid receptors (Joëls, 2008) renders the hippocampus especially vulnerable to chronic stress. Chronic stress elevates glucocorticoid levels, and inhibits synaptic plasticity in the hippocampus, leading to reduced dendritic spine density and reduced synapse numbers, frequently exhibiting notable sex differences (for review see, (Lai et al., 2016)). The optogenetic and chemogenetic activation of the vHIPP-mPFC pathway was necessary and sufficient for ketamine’s antidepressant response (Carreno et al., 2016). Interestingly, chronic pain-induced stress causes an increase in excitatory synaptic transmission from vHIPP pyramidal (CaMK2A) neurons to mPFC corticotropin-releasing hormone-positive (CRH^+^) inhibitory neurons, leading to feed-forward inhibition of mPFC layer V pyramidal neurons (Lv et al., 2024). Thus, chronic stress likely alters the normal PFC-regulated feedback loop such that reciprocal, stress-induced adaptations within the PFC and hippocampus are improperly communicated, ultimately leading to severe, dysregulated circuit-wide feedback.

### The PFC-LHb circuit

The PFC sends direct glutamatergic projections to LHb and regulates aversive information processing (Cerniauskas et al., 2019). In a social fear conditioning paradigm, targeted inhibition of the mPFC–LHb projection resulted in a significant reduction in the magnitude of social fear behaviors (Tian et al., 2024). This suggests that the mPFC uses distinct, pre-specified cellular circuits to process and regulate experiences of differing emotional valences. Utilizing electrophysiology, chemogenetics, and two distinct mouse models of depression, researchers found that reduced extracellular adenosine triphosphate (ATP) levels in the mPFC induced depression-like behavior, and resulted in decreased GABAergic inhibition and elevated excitability specifically in mPFC neurons projecting to LHb, but not dorsal raphe (Lin et al., 2022). Chemogenetic manipulation confirmed this causality: the activation of the mPFC-LH pathway induced depression-like behavior, while its inhibition was sufficient to alleviate behavioral impairments in both susceptible mouse models (Lin et al., 2022). These results provide compelling evidence that mPFC ATP levels critically regulate depressive-like behavior in a molecular- and pathway-specific manner, suggesting a novel mechanism for antidepressant efficacy. Chronic stress induced by the unpredicted, forced loss of social ranking recruits this PFC-LHb pathway. Specifically, *in vivo* fiber photometry and single-unit electrophysiological recordings reveal that forced defeat (but not natural hierarchy loss) produces negative reward prediction error (RPE) in the LHb and inhibits the mPFC, disrupting its ability to regulate social competitiveness in social contests (Fan et al., 2023b). Optical activation of the mPFC or ketamine administration successfully reversed these effects (Fan et al., 2023b). Utilizing chemogenetics and *in vivo* fiber photometry, a recent study showed that the dmPFC actively transmits aversive information to the LHb, displaying increased calcium signaling during stress exposure (Tong et al., 2024). Consequently, the activation of dmPFC-LHb projection and its postsynaptic targets is necessary to drive chronic stress-induced depressive and anxiety-like behaviors (Tong et al., 2024), suggesting this specific pathway may be involved in symptom comorbidity.

### PFC, depression, and neuroinflammation

The projection-specific maladaptations discussed above are profoundly modulated, and often directly driven, by stress-induced neuroinflammation. Consequently, systemic neuroimmune dysregulation may serve as a critical, cross-cutting mechanism that orchestrates the synaptic and circuit-level remodeling observed in MDD. By bridging peripheral immune responses with central nervous system function, inflammation may act as a primary catalyst for dendritic atrophy, neurotransmitter imbalance, and altered plasticity within prefrontal-reward circuitry. Chronic stress paradigms, such as CSDS and CVS initiate a robust neuroinflammatory cascade. This involves microglial morphological and transcriptomic changes even within the PFC (Nie et al., 2018), compromised blood–brain barrier (BBB) integrity, and the mobilization of peripheral immune cells (e.g., monocytes and neutrophils) (Hachenberg et al., 2025). These activated cells then release key pro-inflammatory mediators (e.g., interleukin-6 and matrix metalloproteinase 8) that infiltrate the PFC and limbic brain regions, driving stress-related behavioral pathology (Hachenberg et al., 2025). Chronic stress also activates immune cells to modulate neural circuit activity and remodel synaptic connectivity via synaptic phagocytosis, particularly in the PFC, thereby triggering behavioral deficits (Lee and Wheeler, 2025). Because immune function and its environmental sensitivity exhibit profound sex differences in humans (Bekhbat and Neigh, 2018; Arambula and McCarthy, 2020), this resulting variability in neuroimmunology provides a compelling mechanism to help explain the observed sex-specific variability in the etiology, clinical profile, and treatment progression of MDD. For example, the effects of CSDS on BBB permeability are highly regionally and sex-dependent: female mice show increased BBB permeability in both the PFC and the NAc, while in male mice, this disruption is mainly restricted to the NAc (Menard et al., 2017; Dion-Albert et al., 2022). A subsequent study demonstrated that environmental enrichment and physical exercise can prevent this chronic stress-induced loss of BBB integrity via astrocyte-specific fibroblast growth factor 2 (*Fgf2*) upregulation (Paton et al., 2026). Consequently, novel and existing therapeutics that target immune dysregulation represent highly promising strategies for the treatment of depression. In fact, a recent meta-analysis indicates that anti-inflammatory treatments can safely and effectively mitigate depressive symptoms and anhedonia in depressed individuals with elevated baseline inflammation (Giollabhui et al., 2025). This therapeutic effect could be driven by the targeted inhibition of the IL-4 receptor *α* subunit (IL-4Rα) and the Th2 (T helper 2) axis (He et al., 2026).

### Future direction

Achieving truly personalized and effective treatments for MDD requires a fundamental shift: we need to identify the right treatment for the patient at the right time by precise measurements of behavioral deficits and the underlying neural circuitry (Goldstein-Piekarski and Williams, 2019). Unprecedented progress in artificial intelligence and machine learning now allows for personalized drug rankings for individual patients, marking a significant advance in data-driven psychiatric care and personalized treatment options in MDD (Benrimoh et al., 2025). Advancing brain imaging techniques to achieve unprecedented spatial and temporal resolution will help elucidate the connections between specific symptom phenotypes and *in vivo* neural circuitry. Utilizing these data science and machine learning tools will allow us to reformulate our understanding of the pathophysiology of MDD and other affective disorders, ultimately leading to highly targeted, personalized treatment options.

The behavioral heterogeneity observed across stress-induced models of depression imposes a reconsideration of current classification methods. Relying solely on a simplistic dichotomy, such as categorizing animals as strictly “resilient” versus “susceptible” based on a single behavioral metric or an arbitrary cut-off point, fails to capture the full spectrum of individual variations that emerge following chronic stress (Cerniauskas et al., 2019; Woo et al., 2021; Morel et al., 2024). A novel, comprehensive strategy that accounts for behavioral, molecular, and circuit-level heterogeneity is urgently needed to understand and predict individual variability in stress responses and treatment trajectories.

Finally, the inclusion of sex and gender as fundamental biological variables in the study of MDD and depression-like behaviors must become standard practice. This is indispensable not only due to the significant sex differences observed in disease presentation, progression, and maintenance, but also to maximize our mechanistic understanding and drive the development of effective therapeutics. To achieve this, researchers at all levels must adopt comprehensive methodological standards. This includes utilizing both male and female subjects, explicitly reporting the sex of cell lines and tissues, accounting for sex during randomization or counterbalancing, adequately powering studies to detect sex differences, rigorously detailing experimental and housing conditions, and consistently conducting sex-disaggregated analyses (Hunt et al., 2022; Finn et al., 2026).

## Conclusion

Collectively, the reviewed evidence indicates that chronic, uncontrollable stress impairs prefrontal executive function and dysregulates its cortical and limbic circuitry. By specifically disrupting these downstream reward-processing networks, this stress-induced PFC dysfunction triggers depression-like behaviors. Ultimately, the specific onset and persistence of these stress-induced pathologies vary widely among individuals, meaning that understanding individual susceptibility to depression requires a multimodal and multifactorial framework. The timing and intensity of the stressor interact synergistically with intrinsic biological variables such as age, sex, and genetic predisposition, as well as environmental exposures. These interacting risk factors may also amplify existing functional deficits within the PFC reward system. Given the multifactorial nature of reward circuit dysregulation, future studies should focus on delineating the exact cellular and molecular mechanisms at play within specific prefrontal networks. Elucidating the distinct synaptic changes within the PFC-NAc, PFC-VTA, and PFC-hippocampal projections will be paramount to understanding precisely how prefrontal dysregulation translates into clinical behavioral deficits. Defining the sexually dimorphic trajectories of PFC development, refinement, and stress reactivity remains a critical imperative for ongoing and future investigations. Although clinical vulnerability to depression exhibits clear sex differences, the precise biological underpinnings of this disparity remain critically underexplored. To fully understand these divergent pathways, the field should employ integrated neurobiological approaches, bridging cellular and molecular investigations with systems-level neuroscience, to delineate how maladaptive behaviors develop across both sexes. Finally, investigating the neurodevelopmental trajectories of PFC architecture and function under stressful paradigms is imperative. Such longitudinal studies could illuminate the earliest stages of neuropathology, yield critical biomarkers, and pave the way for preventive interventions prior to circuit-level remodeling.
